# Standardized patients in psychotherapy training and clinical supervision: study protocol for a randomized controlled trial

**DOI:** 10.1186/s13063-020-4172-z

**Published:** 2020-03-18

**Authors:** Franziska Kühne, Peter Eric Heinze, Florian Weck

**Affiliations:** Department of Psychology, Clinical Psychology and Psychotherapy, University of Potsdam, Karl-Liebknecht-Str. 24-25, 14476 Potsdam, Germany

**Keywords:** Clinical psychology, Education, Psychotherapeutic competencies, Psychotherapy research, Role-playing, Simulated patients, Standardized patients, Randomized controlled trial

## Abstract

**Background:**

Psychotherapy is highly effective and widely acknowledged for treating various mental disorders. Nevertheless, in terms of methods for teaching effective psychotherapeutic approaches and competencies, there has been a lack of investigation. Training and supervision are the main strategies for teaching therapist competencies, and standardized role-plays with simulated patients (i.e., trained individuals playing someone with a mental disorder) seem useful for evaluating training approaches. In medical education, this procedure is now internationally established. However, so far, little use has been made of standardized role-playing to evaluate training and supervision in the area of clinical psychology and psychotherapy.

**Methods:**

In this study, standardized role-plays are used to evaluate methods for training and supervision. Central cognitive behavioral approaches for treating depression are taught in the training. The first experiment compares an active training approach (i.e., model learning) with a passive one (i.e., reading manual-based instructions). The second experiment compares a direct supervision technique (i.e., supervision based on video analysis) with an indirect one (i.e., supervision based on verbal reporting). In each experiment, 68 bachelor’s and master’s students of psychology will be randomly assigned to the experimental and control groups. Each student takes part in three role-plays (baseline, post and 3-month follow-up), which are all videotaped. Two independent raters assess therapist competencies in each role-play on the basis of a standardized competence scale.

**Discussion:**

The research project aims to contribute to the development of specific training and supervision methods in order to improve psychotherapy training and patient care.

**Trial registration:**

ISRCTN Registry, ISRCTN19173895. Registered on 10 December 2019.

## Background

Training and supervision of psychotherapists are central to the dissemination of evidence-based psychotherapy [1]. Results on the efficacy of psychotherapy from randomized controlled trials (RCTs) may be integrated adequately into clinical practice only with effective training. To date, psychotherapy has more often been part of research than psychotherapy training; thus, current training approaches cannot be characterized as evidence-based [2, 3].

### Assessment of psychotherapeutic competencies

The appropriate assessment of psychotherapeutic competencies is central to the evaluation of psychotherapy training and clinical supervision methods. Waltz et al. [4] consider therapist competence as the level of skill in treatment delivery, covering relevant contextual variables (e.g., client variables and stage in therapy). For the measurement of competencies, Muse and McManus [5] refer to Miller’s pyramid model [6] and define four levels. At the most basic level of knowledge, measurement may be realized by using multiple-choice questions (MCQs) or essays. For the second level of practical understanding, the former two as well as clinical vignettes and case reports may be used. Assessments of practical understanding may be accomplished via standardized role-plays, and clinical practice assessments via ratings of treatment sessions, supervisory assessments, or patient outcomes. Whereas higher levels of the model are associated more with external validity, lower levels provide more internal validity and opportunities for standardization. Despite their higher external validity, competence assessments within real patient encounters imply a number of difficulties. First of all, competence assessments are dependent on patient difficulty and behavior, and owing to their variability, several measurements are necessary for reliable assessments [7]. By contrast, measuring competence via simulated patients (i.e., at the third level of the pyramid model) reduces the aforementioned problems and facilitates standardization for learning purposes.

### Use of simulated patients

In medical training, the use of standardized role-plays is now widespread [8, 9]. “Simulated patients” are trained persons (laypersons, patients, or actors) portraying a person with a diagnosed disease, whereas “standardized patients” are described as providing their portrayal in a consistent and constant manner [10, 11]. Often, the terms are used interchangeably and are abbreviated as “SP” [9]. In contrast to improvised interactions, SPs are provided with intensive role training in advance of their play, facilitating standardization for training and assessment purposes [11]. Furthermore, SPs may be used for the portrayal of rare clinical situations [12] and to foster experiential learning, modeling, skills training, or learning through feedback [13]. Thus, SPs are commonly used for diagnostics or communication training but also for medical examinations, so-called objective structured clinical examinations [8, 9, 14].

### Simulated patients in clinical psychology

In contrast, within clinical psychology and psychotherapy, the advantages of SPs are not fully exploited and have been recognized only in recent years [15–19]. Although using SPs in clinical psychology is perceived as important for learning, self-efficacy, self-reflection, and therapeutic alliance [15–18], current surveys concentrate on subjective assessments, small samples, or convenience samples or are limited to one-group designs [15–18]. Thus, there is a need for RCTs using SPs in clinical psychology [8].

### Active versus passive strategies

All in all, the evidence indicates that active training strategies (e.g., modeling, feedback, and practical exercises) are more effective than passive ones (e.g., reading manuals) [20]. A meta-analysis by Hill and Lent [21] revealed that training conversational techniques were significantly more effective than no training at all (*d* = 0.89) but that modeling was the most important intervention. But again, most studies were uncontrolled and did not use a training manual, and the meta-analysis did not refer to specific psychotherapeutic interventions (such as cognitive restructuring) but focused on counselling or exploration skills (e.g., showing empathy). Beyond training, competence-based supervision is described as fundamental to the development of psychotherapeutic competencies [22]. Referring to audio or video tapes enables the supervisee to receive specific competence-based feedback, which is considered relevant for skills development [23]. Comparing supervision based on verbal reports (i.e., supervision as usual) with those based on video recordings of therapy sessions, Martino et al. [24] demonstrated that the latter contributed more to competence development. Even so, regarding clinical supervision, only a limited number of systematic and controlled studies have been published to date [12, 25].

### Objectives and hypotheses

Therefore, the aim of this study is to use SPs to evaluate specific training and supervision conditions. Psychology students will be taught distinct interventions relevant for the cognitive behavioral therapy of patients with diagnosed depression (i.e., behavioral activation and cognitive interventions based on an established treatment manual) [26]. The focus on depression was chosen because, on the one hand, it is one of the most prevalent mental disorders [27] and, on the other hand, psychotherapeutic competencies are highly relevant for depression treatment [28, 29].

The study combines the development of SP methods with two consecutive experiments. During the developmental phase, scenarios for the SP portrayals will be developed and validated, instruments for the assessment of therapist behavior and SP authenticity will be developed, SPs will be recruited and trained thoroughly, and their portrayal will be validated on the basis of video recordings.

In the first experiment, two specific training strategies (i.e., reading written instructions with or without modeling) will be compared. In the second experiment, two central supervision techniques (i.e., based on treatment videos versus on verbal reporting) will be evaluated. We expect post-training, psychology students randomly assigned to the active strategy of modeling to display more therapeutic competencies than students who were only reading the written instructions on behavioral activation or cognitive techniques (hypothesis 1). Furthermore, we expect the competence differences between the two groups to persist until the 3-month follow-up (hypothesis 2). Second, we expect that post-supervision, psychology students randomly assigned to supervision based on treatment videos will display more therapeutic competencies than the students who described their treatment verbally to the supervisor (hypothesis 3). Again, we expect the competence differences between the two groups to persist at the 3-month follow-up (hypothesis 4).

## Methods/Design

### Study design

**Fig. 1:**
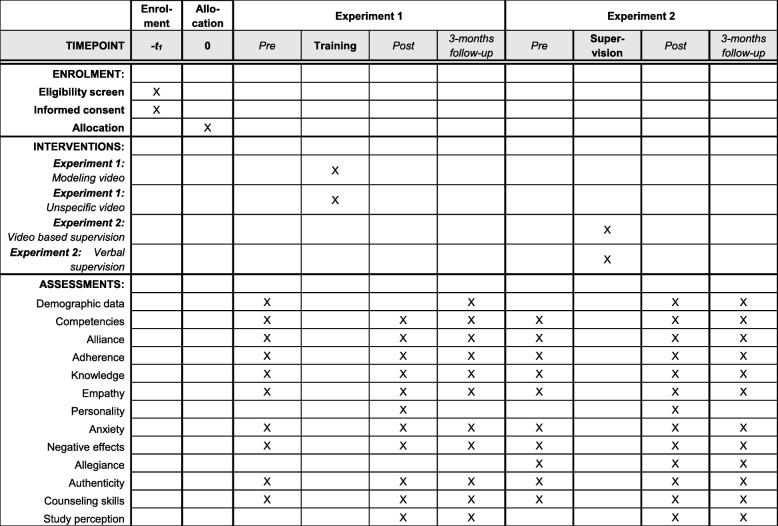
SPIRIT (Standard Protocol Items: Recommendations for Interventional Trials) 2013 figure

The study is a single-center RCT (see Fig. 1) in which participants (i.e., bachelor’s and master’s students included as either trainees or supervisees) will conduct therapy sessions with SPs. All sessions will be video-recorded. Following a preparation period, both experiments will be conducted consecutively within a 2-year period. Data will be gathered before and after training/supervision and at the 3-month follow-up. Participants fulfilling the inclusion criteria (see below) will be randomly assigned by computer-generated numbers in a 1:1 ratio and allocated to the experimental or control groups and to the order of the training topics (behavioral activation and cognitive strategies or vice versa). The allocation will be implemented by a researcher (who is independent of the role-plays). The researcher will enroll and assign participants and provide sealed envelopes containing the study material. To reduce the possibility for unblinding, the researcher and student assistants will urge all participants not to speak to any other students about the trial.

In this educational low-risk trial, there is no formal implementation of a trial steering committee, data monitoring committee, or stakeholder group. Nevertheless, the funder decides upon the interim project report upon further funding. The principal investigators (PIs) are responsible for local organization, which is implemented by the researcher and his student assistants (e.g., regarding recruitment and ensuring informed consent). The PIs and the researcher meet weekly to oversee conduct and progress of the trial. Statistical counseling will be provided by another department. At any time, students may consult the university’s ethics review committee and the data security officer if problems or harms arise. The researcher and student assistants will note down reasons for discontinuation as well as any problems that may arise during the trial (e.g., burdens and stress to the participants). During the weekly consultations with the PIs, these problems will be discussed and decisions will be made on how to proceed (e.g., to provide individual support and to stop the trial).

### Participants

Participants will be recruited from bachelor’s and master’s psychology courses, mainly at the University of Potsdam. They will be included if they have given verbal and written informed consent to the study researchers and therefore agreed to the video recordings of the standardized role-plays. They will be excluded if they are currently in psychotherapeutic treatment or have insufficient German language skills to participate. Participants can withdraw from the study at any time without any disadvantages arising. According to their preference, students will receive either course credit or an expense allowance of €40. Their retention will be promoted by emphasizing the learning benefits of participation. If a reason for discontinuation or unintended effects is given, it will be recorded and, if required, classified into categories. If any impairments through study participation are perceived, those affected will be offered a dialogue with one of the PIs, who are also licensed psychotherapists.

### Standardized patients

The SPs will be recruited at the University of Potsdam from courses other than psychology, such as the educational or natural sciences, in order to reduce the probability that participants and SPs know each other. They will be employed as student assistants in order to ensure their engagement, and they will be blinded to the experimental conditions. The authenticity of their portrayals will be validated beforehand by independent external raters.

### Training intervention and comparator

The first experiment pursues the goal of evaluating the role of modeling for developing therapeutic competencies. The interventions are based on an evidence-based manual for the cognitive behavioral treatment of depression [26].

In the experimental group, participants (i.e., student trainees) will watch a video of an experienced psychotherapist who skillfully demonstrates behavioral activation (video 1) and cognitive (video 2) strategies with an SP demonstrating a depressive disorder. Participants will answer two control questions regarding the contents of the videos as a manipulation check. Additionally, participants will receive written instructions on behavioral activation (instruction 1) and cognitive biases (instruction 2).

In the control group, participants will watch two unspecific learning tutorials (i.e., solving a Rubik’s Cube and folding an origami animal) without psychotherapeutic contents, answer two control questions regarding the contents of the videos, and receive the same written instructions as described above.

### Supervision intervention and comparator

The second experiment will evaluate the role of video-based feedback for developing therapeutic competencies and is based on the concept of competency-based supervision [22]. In the experimental group, participants (i.e., student supervisees) will show their video on behavioral activation (video 1) and cognitive (video 2) techniques of an interaction with an SP demonstrating a depressive disorder to a supervisor. Thus, supervision will be behaviorally based (direct supervision) [30]. In the control group, participants will report their experiences with the two role-plays to a supervisor; thus, the supervision will be verbally based (supervision as usual) [31].

### Duration

Each of the role-plays will last 20 min. Including the pre- and post-measurement, the first appointment will continue for 3.5 h, and the follow-up appointment will last about an hour. Training and supervision interventions are currently not standard elements of clinical psychology courses at German universities but extend beyond the established topics and didactics.

### Supervisors

The supervisors will be licensed psychotherapists (cognitive behavioral therapy). They will be provided with a supervision manual based on Falender and Shafranske [22] and Hautzinger [26]. Supervisors are intended to be recruited from an internal database of former study supervisors. In advance of the first supervision session, they will receive a 10-h training session in order to standardize supervision. Supervisors will not be part of the study team (i.e., they will be external). One supervision session will last 30 min, and each supervisor will receive €50.

### Raters

The two raters will be licensed psychotherapists or therapists-in-training (cognitive behavioral therapy) and are also intended to be recruited from an internal database of former raters. In advance of the first session, they will receive 10 h of training on the use of the instruments for the assessment of the video-recorded interactions. Raters will not be part of the study team and will conduct their ratings independently of each other. They will be blinded to the experimental conditions. As a manipulation check, they will be asked at the end of each rating whether they can identify the condition (experimental versus control). One assessment will take around 30 min, and each rater will receive €20.

### Primary outcome

Psychotherapeutic competencies serve as the primary outcome. They will be assessed by trained raters (see above) via the German adaptation [32, 33] of the Cognitive Therapy Scale [34]. Therapeutic techniques will be assessed by the raters using a self-developed therapeutic skills checklist based on published instruments [35–39] and adapted to the psychotherapy field.

### Secondary outcomes

Therapeutic alliance and the participant empathy will be evaluated by the raters, participants, and SPs. We will use the Helping Alliance Questionnaire [40], or its German version [41], and the Empathy Scale [42], which will be adapted to the three perspectives.

For measuring therapeutic adherence, we will use the Cognitive Behavioral Therapy Adherence Scale (CBT-AS) [43]. The CBT-AS will be adapted for the topics of behavioral activation and of cognitive biases based on the depression manual used [26].

The therapeutic knowledge of participants will be evaluated by MCQs and case vignettes [5]. The materials will be self-developed in accordance with the above-mentioned manual [26] and recommendations for the construction of MCQs [43].

Further outcomes taken into consideration will be the participant’s personality (Big Five Inventory, or BFI-K) [44], state–trait anxiety (State–Trait Anxiety Inventory) [45], and individual perception of the study adapted from [46, 47].

The raters will evaluate the SP’s authenticity (Ay DS, Kühne F, Weck F: Can simulated patient encounters appear authentic? Development and pilot results of an observer-based rating instrument, submitted), and the SPs will evaluate the participant’s counseling skills [42, 48].

Negative therapist effects will be assessed by items adapted from published German questionnaires [49, 50]. Furthermore, possible allegiance effects of supervisors will be captured by a questionnaire adapted to supervision [51].

### Statistical analysis and power calculation

To avoid attrition bias, we will include all randomly assigned participants in the primary analyses (intention-to-treat). Second, we will perform per-protocol analyses and compare the results with those from the intention-to-treat analyses. We will use chi-squared tests for nominal data and analyses of variance/analyses of covariance (ANOVAs/ANCOVAs) for ordinal and interval data. For the analysis of training/supervision effects, the ANOVAs will involve a group factor (training/supervision condition) and a time factor (before, after, and follow-up). Possible pre-differences between the groups will be taken into account via the ANCOVAs. The α level will be set at 0.05 and Bonferroni-corrected for multiple testing. Missing data will be addressed according to the outcome and data structure (e.g., complete case analyses and imputation by expectation-maximization or by last observation carried forward).

Considering prior studies [24, 52], we assume moderate competence differences between the groups (Cohens *d* = 0.60) and high correlations between competence ratings (*r* = 0.50). Given an *α* of 0.05 and a 1-*β* of 0.80 (power), a sample of 68 participants will be necessary in each experiment, resulting in an overall sample size of 136 (G*Power Version 3.1.9.2) [53].

## Discussion

This RCT combines the investigation of specific training and supervision techniques in clinical psychology by using SPs and a validated observer-based rating scale for the assessment of psychotherapeutic competencies. We thus expect the study to contribute to evidence-based education and training in the treatment of mental disorders [1]. Developing SP scenarios for the field of clinical psychology, gathering knowledge in the use of SPs, and assessing competencies within standardized encounters will contribute to further developments within our field.

SPs enable approximating clinical encounters but may not replace them. Therefore, external validity will be lower in this study than in patient studies. The legal regulations for qualification and licensure of psychotherapists are about to be reformed by the German Federal Ministry of Health. In the draft law, practical education is extended even within the master’s program [54], which will require evidence-based knowledge on effective training strategies. Above and beyond that, professional bodies such as the American Psychological Association [55] require obtaining and enhancing therapeutic competencies during profession-long learning. Therefore, the results might contribute to the development of licensed therapists and thus to the effective treatment of mental health patients.

## Trial status

This is the first protocol version (10 December 2019). Recruitment of participants started on 4 December 2019 and will end when a complete sample size is attained (around 31 December 2021). If protocol modifications prove necessary, we will communicate them within the study publications and the final report to the funder.

## Abbreviations

ANCOVA: Analysis of covariance
ANOVA: Analysis of variance
CBT-AS: Cognitive Behavioral Therapy Adherence Scale
MCQ: Multiple-choice question
PI: Principal investigator
RCT: Randomized controlled trial
SP: Simulated/standardized patient
